# Recommendations for a uniform assessment of publication bias related to funding source

**DOI:** 10.1186/1471-2288-13-120

**Published:** 2013-09-30

**Authors:** Marlies van Lent, John Overbeke, Henk J Out

**Affiliations:** 1 Clinical Research Centre Nijmegen, Department of Pharmacology – Toxicology, Radboud University Nijmegen Medical Centre, PO Box 9101, 6500 HB, Nijmegen, The Netherlands; 2 Department of Primary and Community Care, Radboud University Nijmegen Medical Centre, PO Box 9101, 6500 HB, Nijmegen, The Netherlands; 3 Teva Pharmaceuticals, Computerweg 10, 3542 DR, Utrecht, The Netherlands

**Keywords:** Publication bias, Clinical drug research, Sponsorship, Pharmaceutical industry, Trial outcomes, Recommendations, Uniform assessment

## Abstract

**Background:**

Numerous studies on publication bias in clinical drug research have been undertaken, particularly on the association between sponsorship and favourable outcomes. However, no standardized methodology for the classification of outcomes and sponsorship has been described. Dissimilarities and ambiguities in this assessment impede the ability to compare and summarize results of studies on publication bias. To guide authors undertaking such studies, this paper provides recommendations for a uniform assessment of publication bias related to funding source.

**Methods and results:**

As part of ongoing research into publication bias, 472 manuscripts on randomised controlled trials (RCTs) with drugs, submitted to eight medical journals from January 2010 through April 2012, were reviewed. Information on trial results and sponsorship was extracted from manuscripts. During the start of this evaluation, several problems related to the classification of outcomes, inclusion of post-hoc analyses and follow-up studies of RCTs in the study sample, and assessment of the role of the funding source were encountered. A comprehensive list of recommendations addressing these problems was composed. To assess internal validity, reliability and usability of these recommendations were tested through evaluation of manuscripts submitted to journals included in our study.

**Conclusions:**

The proposed recommendations represent a first step towards a uniform method of classifying trial outcomes and sponsorship. This is essential to draw valid conclusions on the role of the funding source in publication bias and will ensure consistency across future studies.

## Background

### Introduction

In recent years, the issue of publication bias in clinical drug research has been widely addressed. Studies reporting results that are positive or favourable to the experimental drug are more likely to be published, and outcomes that are statistically significant have higher odds of being fully reported [1-3]. Funding by the pharmaceutical industry and financial conflicts of interest among authors have particularly been associated with publication of favourable outcomes [4-6]. Publication bias could result from researchers and sponsors failing to submit trials with negative results, or from favouritism towards publication of positive results among peer reviewers, journal editors and publishers.

Numerous studies on the association between sponsorship and trial outcomes have been undertaken [6]. Classification of outcomes as positive or negative and establishing the role of the funding source may appear to be straightforward. However, when reviewing studies on publication bias, no standardized methodology for the assessment of outcomes and sponsorship has been described. Definitions used for classification of trial outcomes are inconsistent across studies and the terms ‘sponsored by industry’ and ‘conflict of interest’ have been applied in different ways.

In a majority of the studies on publication bias, methods for classification of trial outcomes are only reported to a very limited extent, though there are positive exceptions [5,7]. However, this assessment is not without hurdles, for instance when reviewing results of non-inferiority or equivalence trials and trials reporting safety rather than efficacy outcomes. Some studies concentrate on results reported for primary endpoints, while others examine conclusion sections to classify outcomes. Some investigators briefly mention that included trials are categorized according to funding source [7-9], while in other papers explicit criteria are given for the classification based on sponsor type. Drug trials are often considered to be industry-sponsored when funded by a pharmaceutical company [6,10-12], while some researchers also regard donation of study medication by a manufacturer as industry-sponsorship [6,10,11,13]. In addition, drug trials are sometimes classified as industry-sponsored if authored by one or more employees of the company manufacturing the test drug [10,11,13,14]. Furthermore, the terms industry-funded, industry-supported, and industry-sponsored are used interchangeably when categorizing trials according to sponsorship.

Dissimilarities and ambiguities in the classification of outcomes and sponsorship impede the ability to compare and summarize results of studies on publication bias. A standardized approach, taking into account different types of financial and material support and a clear definition of trial outcomes, is essential to draw valid conclusions on the role of the funding source in publication bias. To guide authors undertaking studies on the association between sponsorship and study outcomes or authors undertaking systematic reviews or meta-analyses of such studies, this paper provides recommendations for a uniform assessment of publication bias related to funding source.

## Methods

As part of ongoing research into biased reporting of trial outcomes, we assessed the role of editorial and peer review processes in publication bias. We evaluated whether submitted manuscripts with negative outcomes were less likely to be published than studies with positive outcomes, and determined the influence of sponsorship in this context. We retrospectively reviewed manuscripts submitted to eight journals from January 2010 through April 2012. One general medical journal (British Medical Journal) and seven specialty journals (Annals of the Rheumatic Diseases, British Journal of Ophthalmology, Diabetologia, Gut, Heart, Journal of Hepatology, and Thorax) were included. Journals were selected based on impact factor (journals indexed with the highest impact factors within subject categories, according to Institute for Scientific Information Journal Citation Report 2011), and the number of drug RCTs published in 2010–2011. Manuscripts reporting results of RCTs were included, if at least one study arm assessed the efficacy or safety of a drug intervention and a statistical test was used to evaluate treatment effects (n = 472). Two authors (MvL, HJO) extracted information on trial results and financial or material support from manuscripts.

During the start of this evaluation, we encountered several problems related to (1) the classification of outcomes, (2) inclusion of post-hoc and subgroup analyses and follow-up studies of RCTs in the study sample, and (3) assessment of the role of the funding source. These problems hampered unequivocal judgment on sponsorship and the direction of trial outcomes. We composed a list of recommendations addressing these problems, to facilitate a standardized assessment of manuscripts included in our study and future studies on publication bias. To assess internal validity, reliability and usability of the recommendations were tested through evaluation of manuscripts submitted to journals included in our study. Minor adjustments were made during this process, and a comprehensive list of recommendations was created.

## Findings

### Identified problems

#### Classification of trial outcomes

##### Poorly described methodology and lack of (or multiple) primary endpoints

A poor description of trial design in research papers complicates the selection of manuscripts that are eligible for inclusion when conducting a study on publication bias. As trial outcomes are often classified based on results for reported primary endpoints, failure to accurately define a primary endpoint among a plurality of outcomes described or mentioning multiple primary endpoints constitutes a problem. Guidelines for accurate reporting of RCTs are available by means of the CONSORT Statement (Consolidated Standards of Reporting Trials) [15], but the quality of reporting regarding these and other aspects remains sub-optimal after publication of CONSORT recommendations [16,17].

##### Safety as primary endpoint

An issue not related to the adequacy of reporting, but to the type of outcomes reported, is represented by trials with safety rather than efficacy as a primary endpoint. It could be argued whether trial results should be classified as positive when the test drug is proven to be as safe as the control treatment, or whether this would only be valid when less adverse events are reported for the test drug than for the comparator. Furthermore, it can be difficult to determine whether reported outcomes should be considered as efficacy or safety endpoints.

##### Non-inferiority and equivalence trials

Problems related to trial design also apply to outcomes of non-inferiority and equivalence trials. In case non-inferiority or equivalence is established for treatments in these trials, outcomes could be considered positive, as they confirm the hypothesis of the trial. However, there are no clinically relevant differences between treatments with regard to the primary outcome of the trial, which could also be interpreted as a negative finding.

#### Inclusion of post-hoc analyses and follow-up studies of trials

It could be argued whether post-hoc and subgroup analyses and follow-up studies of RCTs should be included in studies on publication bias, next to original trial reports. Due to inadequate reporting on methodological aspects as described above, it can be difficult to discriminate between original reports and secondary analyses of trial data based on publications in the scientific literature. Unless original trial protocols are available, it is often not possible with a high degree of certainty to make judgments on what is post-hoc and what is not. However, post-hoc and subgroup analyses and follow-up studies of RCTs could be equally prone to influences of sponsorship and direction of results on publication of studies.

#### Role of the funding source

##### Different levels of support

Assessment of the role of the funding source in drug trials is complex because of the variety of levels of involvement reported, ranging from financial support and donation of study medication by a pharmaceutical company, to input of the manufacturer in the trial design, conduct, data analysis and publication of results. Financial support from industry is often described as unrestricted educational grants, which suggests that funding was given without any predefined involvement of the funder. Furthermore, some trials are authored by one or more employees of the company manufacturing the test drug or funding the study.

##### Inadequate reporting of sponsorship

The assessment of sponsorship can be complicated by the fact that some journals do not report funding sources or do not explain the sponsor’s role in a trial, and authors may not disclose received funding. Consequently, trials receiving industry funding could be falsely classified as non-industry. The CONSORT 2010 Statement and the Uniform Requirements for Manuscripts Submitted to Biomedical Journals of the International Committee of Medical Journal Editors (ICMJE) [15,18], both adopted by many journals, insist on reporting of information on funding sources and other support and the role of funders. However, not all authors adhere to these guidelines when submitting manuscripts, and even if authors do adhere to them, journal editors may not require the material to be reported.

##### Formal sponsorship

It is often unclear based on the information reported in manuscripts whether trials should be regarded as investigator-initiated studies or that pharmaceutical companies qualify for formal sponsorship as defined in the ICH-GCP (International Conference on Harmonisation of Technical Requirements for Registration of Pharmaceuticals for Human Use – Good Clinical Practice) guidelines [19]. According to these guidelines, a study sponsor is “an individual, company, institution, or organization which takes responsibility for the initiation, management, and/or financing of a clinical trial.” Therefore, financial support only does not necessarily imply formal sponsorship by the donor. In the public trial registries ClinicalTrials.gov, EU Clinical Trials Register and ISRCTN Register, definitions for funding and sponsorship are not consistent across registries and do not unequivocally discriminate between trials formally sponsored by industry according to ICH-GCP guidelines, and those receiving only financial or material support.

##### Non-profit trials

When absence of industry support is evident, it can subsequently be difficult to distinguish between trials sponsored by governmental sources versus other nonprofit organisations. Frequently, it is unclear which party carries responsibility regarding quality and safety measures during trials. Furthermore, trials can be supported by non-industry organisations, that are owned by pharmaceutical companies.

### Recommendations

#### Classification of trial outcomes

Outcomes of drug RCTs should be classified as *positive* if results for the primary endpoint (as reported) are statistically significant (p < 0.05 or 95% confidence interval [CI] for difference excluding 0 or 95% CI for ratio excluding 1) and supports the efficacy of the test drug; or *negative* if the result does not reach statistical significance or is statistically significant in the direction of the control treatment being more efficacious (Table 1). When a trial is designed as an equivalence or non inferiority study and the two treatments are equivalent, i.e. differences in treatment effects do not equal or exceed preset margins, results should be classified as *positive*. When treatments are equally effective regarding the primary endpoint and it is not clearly stated whether a trial was designed as superiority or non-inferiority trial, results should be classified as *positive*. For manuscripts describing a safety parameter as primary endpoint, trials in which the test drug is reported to be as safe or safer than the control treatment (either placebo or active comparator) should be considered as *positive*. When authors explicitly hypothesized that the test drug is expected to be less harmful than control, only trials reporting results that support the safety of the test drug should be classified as *positive*. If treatments are equally harmful, results should be classified as *negative*.

**Table 1:** Classification of outcomes of drug RCTs based on results reported for primary endpoints

	**Positive outcome**	**Negative outcome**
Results for primary endpoint statistically significant and supporting the efficacy of test drug	**X**	
Results for primary endpoint do not reach statistical significance		**X**
Results for primary endpoint statistically significant in direction of control treatment being more efficacious		**X**
Treatments equivalent regarding primary endpoint in non-inferiority or equivalence trials	**X**	
Treatments equally effective regarding primary endpoint in trials not explicitly described as superiority or non-inferiority study	**X**	
Test drug as safe or safer than control treatment in trials with safety parameter as primary endpoint	**X**	
Treatments equally harmful in trials with safety parameter as primary endpoint, when hypothesized that test drug is expected to be safer than control		**X**
Results for >50% of (primary) endpoints statistically significant in favour of test drug, when no/multiple primary endpoints are reported	**X**	
Results for one primary endpoint statistically significant in favour of test drug, when two co-primary endpoints are reported		**X**
Treatment effects not compared between groups but against baseline in each arm; results for primary endpoint statistically significant in favour of test drug	**X**	

If no primary outcome is stated for a trial, or multiple primary endpoints are selected, results should be classified based on the statistical significance and direction of most (primary) outcomes (>50%). When two co-primary outcomes are stated, results for both endpoints should support the efficacy or safety of the test drug in order to classify a trial as *positive*. If results for only one of the two endpoints are statistically significant in favour of the test drug, results should be classified as *negative*. When treatment effects are not directly compared between groups in the statistical analysis (i.e. against baseline measurements in each treatment arm), results should be classified as *positive* if they are statistically significant in favour of the test drug. If uncertainty remains over the classification of trial outcomes, studies should be scored as *negative*, unless the overall tone of the conclusion section favours the test drug.

#### Inclusion of post-hoc analyses and follow-up studies of trials

Studies explicitly referred to as post-hoc or subgroup analyses and follow-up studies of single RCTs are eligible for inclusion in studies on publication bias, if they compare the efficacy or safety of treatments to which participants have been assigned in an RCT, but with regard to other (secondary) outcomes, limited to a specific part of the original study population, or with an extended follow-up in these analyses. Post-hoc and subgroup analyses based on combined data of multiple RCTs should not be included.

#### Role of the funding source

Drug trials should be considered as *non-industry* studies when no associations with a pharmaceutical company can be identified in the trial manuscript. No author-industry affiliations are reported, no manufacturer is named in the materials and methods section or acknowledgements for providing study medication or placebos free of charge, and no statements are made about financial support from pharmaceutical companies (Table 2). These RCTs are sponsored by governmental or other non-profit organizations, including universities, hospitals and foundations.

**Table 2:** Classification of drug RCTs according to sponsor type

		**Non-industry trial**	**Industry-supported trial**	**Industry-sponsored trial**
1.	Pharmaceutical company explicitly reported as study sponsor in manuscript			**X**
2.	Company funding the trial participated in design, conduct, analysis, writing of article and/or decision to publish			**X**
3.	Trial funded by industry and author(s) affiliated to industry, but role of funding source not reported			**X**
4.	Financial support received from pharmaceutical company		**X**	
5.	Donation of study medication or placebos by manufacturer		**X**	
6.	One or more authors employed by pharmaceutical company manufacturing the test drug		**X**	
7.	Trial supported by nonprofit organisation owned by pharmaceutical company		**X**	
8.	Investigator-initiated post-hoc analysis of single RCT formally sponsored by industry		**X**	
9.	Pharmaceutical industry not in any way involved in trial	**X**		
Formal study sponsor according to ICH-GCP guidelines		Nonprofit organisation	Nonprofit organisation	Pharmaceutical company
Responsible party for quality and safety measures during trial		Nonprofit organisation	Nonprofit organisation	Pharmaceutical company

Trials should be classified as *industry-sponsored* when a pharmaceutical company is explicitly described as study sponsor in the manuscript, or when the company funding the trial is reported to have participated in the study design, data collection, analysis, preparation of the manuscript, and/or decision to publish. Involvement of authors employed by a pharmaceutical company is indicative of industry sponsorship, but not sufficient to qualify a trial as industry-sponsored. Studies reporting donation of study medication or receipt of financial support from a pharmaceutical company, and trials with one or more authors employed by the company manufacturing the test drug should be assigned to a separate class of *industry-supported* trials. Sole provision of grants or drugs does not fully comply with criteria for sponsorship according to ICH-GCP guidelines, and thus differs from formal sponsorship by a pharmaceutical company. However, when a trial is funded by industry and there are company-employed co-authors, the company will likely participate in the conduct and reporting of the trial. These trials should be classified as *industry-sponsored*, if the role of the funding source is not explicitly reported. Trials funded or otherwise supported by a nonprofit organisation owned by a pharmaceutical company should be categorized as *industry-supported*. Investigator-initiated post-hoc analyses of single RCTs that were formally sponsored by industry should also be scored as *industry-supported*. When doubt remains over sponsorship and the trial is registered, information in the trial registry should take precedence over other sources of information.

## Discussion

In this study, several problems related to the classification of outcomes and sponsorship were identified. We have formulated recommendations addressing these problems to facilitate a uniform assessment of manuscripts included in studies on publication bias.

In this article, we have focused on the assessment of publication bias related to funding source. However, the relationship between favourable outcomes and sponsorship is much more complex. Numerous systematic reviews have found that industry sponsorship is associated with results that are favourable to the product of the company funding the trial [4,20,21]. Besides publication bias, there are other ways by which outcomes may be influenced. Industry bias may occur through the choice of comparators, dosing and timing of comparisons, coding of events and selective data analysis, interpretation of data and selective outcome reporting [6,21,22]. Although we have concentrated on publication bias, our recommendations are generally applicable to studies comparing industry versus non-industry trials in relation to favourable outcomes. Furthermore, we only considered drug RCTs, but the recommendations are equally relevant to device studies, as these are also often sponsored by companies with a financial interest in the study outcomes.

In our recommendations, reported results are classified as positive or negative. Trials in which primary endpoints are reached or study hypotheses are confirmed are considered to be positive. Possible alternatives to categorize trials would include ‘significant’ vs ‘non-significant’, or ‘favorable’ vs ‘unfavorable’ findings. However, use of these terms would impede the classification of non-inferiority or equivalence trials and studies with safety parameters as the primary endpoint. We have not included a third ‘neutral’ category, as we aimed to keep the classification as straightforward as possible. Moreover, previous studies on publication bias found relatively low numbers of studies with neutral or unclear results [23-25], so we included these in the ‘negative’ category.

For the classification of outcomes, we have concentrated on results reported for the primary endpoint. However, in several studies on industry bias, trials are categorized as positive if the conclusion is favourable (i.e. the authors recommend the test drug), not the reported results [13]. In this article, we recommend considering conclusions only when trials cannot be classified based on reported results. Previous studies reporting on concordance between study results and conclusions found that industry-sponsored trials were less concordant than non-industry studies [6]. This lack of concordance, and the finding that industry-sponsored studies are more likely to have favourable conclusions [6], may be explained by the use of distorted presentation or ‘spin’ in papers [26]. As results may be more objective measures of treatment effects, we chose to concentrate on the assessment of results. Future studies on publication bias should clearly state whether trial results or conclusions are being examined.

Trials were classified as non-industry, industry-supported or industry-sponsored. One of the most fundamental differences between industry-supported and industry-sponsored studies relates to the overall responsibility for the conduct of the trial as defined by ICH-GCP guidelines. Industry-supported trials are done under the responsibility of non-profit organizations in contrast to industry-sponsored studies. This distinction is usually not made in previous literature but is very relevant in terms of quality assessment of reported research. We have not included a category of ‘sponsorship not stated’. Many journals nowadays ask for information on received funding and the role of the funding source, and require that manuscripts on RCTs conform to ICMJE requirements and CONSORT guidelines. Therefore, most trials recently submitted should report on funding and be registered in a trial registry, making information on sponsorship traceable. However, the non-industry category may include trials that received industry support, if authors failed to disclose funding. In a recent Cochrane review on sponsorship and research outcomes, studies were also coded as non-industry if it was not reported who sponsored the study. The review authors stated that some of these trials were likely industry-sponsored, but no changes in results were seen when studies without sponsorship statements were excluded [6]. In addition, industry-sponsored studies may be misclassified as industry-supported, as apparently independently conducted industry-supported trials may have unreported sponsor involvement [27].

For future studies on publication bias, it is important that the quality of reporting on design and sponsorship of trials is improved. Journals should actively enforce the CONSORT Statement and ICMJE requirements, and could require authors to complete a form for disclosure of sponsorship and support received for the reported trial [28].

We emphasize that our recommendations are only validated for use by three investigators. In order to prove whether the recommendations are acceptable to researchers in general, it is essential that the external validity of this proposed classification system is determined in a study undertaken by different researchers on a different sample of manuscripts. To establish external validity, a Delphi consensus technique could be conducted among a larger group of researchers. Future research should focus on whether the classification system is adopted in practice by investigators undertaking studies on trial outcomes and sponsorship.

In conclusion, the recommendations proposed in this article represent a first step towards a uniform method of classifying trial outcomes and sponsorship. This is essential to draw valid conclusions on the role of the funding source in publication bias and will ensure consistency across future studies.

## Abbreviations

CI: Confidence interval; CONSORT: Consolidated standards of reporting trials; ICH-GCP: International conference on harmonisation of technical requirements for registration of pharmaceuticals for human use - good clinical practice; ICMJE: International committee of medical journal editors; RCT: Randomised controlled trial.

## Competing interests

All authors have completed the ICMJE uniform disclosure form at http://www.icmje.org/coi_disclosure.pdf (available on request from the corresponding author). HJO is a paid employee from Teva Pharmaceuticals next to his professorship at the university; JO is the immediate past-president of the World Association of Medical Editors (WAME). MvL reports no competing interests.

## Authors’ contributions

The ICMJE criteria for authorship are read and met by MvL, JO and HJO. HJO originated the idea for this manuscript with support from MvL and JO. MvL wrote the first draft of the paper. The majority of the research underpinning the paper was undertaken by MvL. JO has reviewed and revised the article together with HJO and MvL. All authors agree with the manuscript’s results and conclusions and approved the final version submitted for publication. HJO is the guarantor of this article.

## Pre-publication history

The pre-publication history for this paper can be accessed here:

http://www.biomedcentral.com/1471-2288/13/120/prepub
